# Comparison of the Effects of Blood Cardioplegia and Del Nido Cardioplegia on Postoperative Intensive Care Needs, Drainage, and Renal Functions in Patients Undergoing Isolated Coronary Artery Bypass

**DOI:** 10.21470/1678-9741-2024-0237

**Published:** 2025-05-28

**Authors:** Yaşar Sarıgol, Serkan Yıldırım, Mehmet Işık, Omer Tanyeli, Yuksel Dereli, Erdal Ege, Niyazi Gormuş

**Affiliations:** 1 Department of Cardiovascular Surgery, Necmettin Erbakan University Faculty of Medicine Hospital, Konya, Turkey

**Keywords:** Surgery, Cardiopulmonary Bypass, Acute Kidney Injury, Cardioplegia, Induced Heart Arrest, Constriction, Drainage

## Abstract

**Objective:**

A variety of cardioplegia techniques with different components are implemented to ensure myocardial protection, in addition to keeping the operationa field immobile and free of blood during cardiac surgery. The implemented cardioplegia has unwanted negative effects on other end organs. In this study, our aim was to compare the effects of Del Nido cardioplegia and blood cardioplegia solutions on postoperative intensive care duration, drainage, and renal functions for patients undergoing cardiopulmonary bypass and bypass graft operations.

**Methods:**

Selections were made from patients undergoing elective bypass graft operations in our clinic from January 1, 2022 to December 31, 2023. Patients were randomly selected, retrospectively assessed, and divided into two groups — De Nido group (Group 1) and blood cardioplegia group (Group 2). Comparisons were made between these groups in terms of intensive care duration, drainage, and renal functions.

**Results:**

The study included 120 patients. The Del Nido cardioplegia group included 60 patients, with 60 patients in the blood cardioplegia group. Comparisons between the groups found that the aortic cross-clamping duration was significantly high in Group 1 (*P* = 0.014). The noradrenaline dose given to Group 1 was high (*P* = 0.004). In terms of renal injury, significant degree of elevation was present in Group 1 (*P* = 0.027). The longer aortic cross-clamping duration in Group 1 may be assessed as a determinant factor for noradrenaline dose and acute kidney injury.

**Conclusion:**

This study concluded that it willbe appropriate to choose the cardioplegia method by performing broader meta-analysis studies and minimizing limiting factors.

## INTRODUCTION

For effective and successful cardiac surgery, the desire is for an immobile and blood-free surgical field along with continued perfusion of cardiac myocardial tissue at basal levels. The most important method for myocardial protection is to implement cardioplegia^[1^, ^2]^. Stopping the heart in diastole with hyperkalemic solutions reduces oxygen consumption of the heart, in addition to providing an immobile and blood-free environment^[3^, ^4]^. With this aim, a variety of cardioplegia techniques with different components are used. With cardioplegia, the desire is to ensure maximum myocardial protection without negative effects, or with minimum effect, on other organs. Efforts to find the ideal cardioplegia are among the important research areas in cardiovascular surgery.

After inadequate myocardial protection during open heart surgery, morbidities may occur. As a result, the patient may require longer hospital or intensive care stay, and this leads to increasing drug use, treatment costs, and loss of labor, which creates a public health problem^[5]^.

Developing after open heart surgery, acute renal disease and chronic kidney disease are related to increasing morbidity and mortality^[6^, ^9]^. In adults, mostly single-dose Del Nido cardioplegia (DNC) and multiple-dose blood cardioplegia (BC) are used in surgical practice. In our study, we aimed to assess the effects of DNC and BC used in patients on postoperative intensive care duration, drainage, and kidney functions.

## METHODS

This was a retrospective study. Patients were chosen among those undergoing elective bypass in our clinic from January 1, 2022 to December 31, 2023. The study began after receiving ethics committee permission (2023/4416). Exclusion criteria were patients younger than 18 years, emergency cases, reoperation patients, those with accompanying cardiac surgery, pregnant and breastfeeding women, those with cancer, with acute or chronic kidney failure, bleeding dyscrasia, whose file and document information could not be accessed, with ejection fraction < 30, with cardioplegia other than DNC and BC, with proximal anastomosis accompanying aortic cross-clamping (ACC), and with graft anastomosis with numbers different than three coronary grafts. The study included 120 patients abiding by our study criteria. Patient data were obtained from the archive files in the hospital and daily monitoring records in the intensive care unit (ICU). Demographic, preoperative, operative, and postoperative data were recorded. Patients were divided into two equal groups. These were patients given DNC (Group 1) and patients given BC (Group 2). During the preoperative 24-hour period, patient blood serum creatinine, blood urine nitrate (BUN), and glomerular filtration rate (GFR) values were recorded. On the postoperative first, second, third, and fourth days, blood laboratory results, serum creatinine, BUN, and GFR were examined, and 24-hour drainage and urine output were investigated in the postoperative first and second 24-hour periods. According to Acute Kidney Injury Network criteria, patients were divided into acute renal failure (ARF) stages (stages 1, 2, and 3).

### Cardioplegia Techniques

The DNC group was given 20 ml/kg dose cardioplegia to ensure cardiac arrest. Patients with ACC duration > 60 minutes were given an additional 10 ml/kg DNC dose. The solution was prepared with Isolyte® S 1000 mL (four units of solution + one unit of blood, pH 7.4) + mannitol (20%) 17 ml + magnesium (15%) 14 ml + NaHO_3_ (1 mEq-ml) 13 ml + KCl (2 mEq-ml) 26 ml + lidocaine (2%) 6.5 ml. In our clinic, due to difficulty accessing Plasma-Lyte A, Isolyte® S is used.

Patients in the group with BC had 500 mL isotonic 0.9% solution + KCl (1 mEq-ml) 40 ml + MgSO_4_ (15%) 30 ml + NaHCO_3_(1mEq-ml) 10 ml prepared. Initial administration to our patients used 10 ml/kg dose given at speeds of 250-300 ml/min. Then at 15- to 20-minute intervals, 3-4 ml/kg maintenance doses were administered.

### Statistical Analysis

Data obtained as a result of the research were analyzed with the IBM Corp. Released 2023, IBM SPSS Statistics for Windows, version 29.0.2.0, Armonk, NY: IBM Corp. program. For descriptive analyses, frequency data are given as number (n) and percentage (%), while numerical data are given as mean ± standard deviation or median (first quartile-third quartile) and minimum-maximum. Normal distribution of data was analyzed with visual (histogram, Q-Q plot) and analytical (Kolmogorov-Smirnov, Shapiro-Wilk) methods. Comparison of numerical data between two independent groups used the independent groups f-test for data with normal distribution and the Mann-Whitney U test for data without normal distribution. Comparisons of categorical data used the chi-square (χ^2^) or Fisher’s exact chi-square test. Significance was assessed at *P* < 0.05 level.

## RESULTS

The study included a total of 120 patients, with 60 undergoing DNC and 60 undergoing BC. The mean weight of patients with DNC was 79.25 ± 12.83 kg, mean age was 60.23 ± 9.23 years, and mean body surface was 1.86 ± 0.22 cm^2^. For patients with BC, mean weight was 80.08 ± 11.54 kg, mean age was 62.92 ± 8.65 years, and mean body surface was 1.90 ± 0.15 cm^2^. Of patients with DNC, 48 were men (80%), 12 were women (20%), 30 had diabetes mellitus (DM) (50.0%), 25 had hypertension (HT) (41.7%), 60 were given cardioplegia once (100%), mean preoperative urea was 33.33 ± 10.00, mean preoperative creatinine was 0.86 ± 0.15, and mean preoperative GFR was 94.17 ± 19.96. For patients given BC, 46 were men (76.7%), 14 were women (23.3%), 32 had DM (53.3%), 26 had HT (43.3%), none were given cardioplegia once, 15 were given it twice (25.0%), 39 were given it three times (65.0%), and six were given cardioplegia four times (10.0%), mean preoperative urea was 33.02 ± 10.39, mean preoperative creatinine was 0.91 ± 0.15, and mean preoperative GFR was 84.99 ± 14.99. In terms of preoperative GFR, patients undergoing DNC were found to be statistically significantly different (P = 0.005) (Table 1).

**Table 1:** Comparison of some preoperative and operative values according to the applied cardioplegia method.

Features	DNC (n = 60)	BC (n = 60)	*P*-value
Weight (kg), mean ± SD	79.25 ± 12.83	80.08 ± 11.54	0.709
Age (years), mean ± SD	60.23 ± 9.23	62.92 ± 8.65	0.103
Body surface area, mean ± SD	1.86 ± 0.22	1.90 ± 0.15	0.301
Male, n (%)	48 (80.0%)	46 (76.7%)	-
Female, n (%)	12 (20.0%)	14 (23.3%)	-
DM, n (%)	30 (50.0%)	32 (53.3%)	-
No DM, n (%)	30 (50.0%)	28 (46.7%)	-
HT, n (%)	25 (41.7%)	26 (43.3%)	-
No HT, n (%)	35 (58.3%)	34 (56.7%)	-
Number of cardioplegia administrations, n (%)	60 (100%)	60 (100%)	-
Given once, n (%)	60 (100%)	0 (0.0%)	-
Given twice, n (%)	0 (0.0%)	15 (25.0%)	-
Given 3 times, n (%)	0 (0.0%)	39 (65.0%)	-
Given 4 times, n (%)	0 (0.0%)	6 (10.0%)	-
Preoperative urea, mean ± SD (mg/dL)	33.33 ± 10.00	33.02 ± 10.39	0.870
Preoperative creatinine, mean ± SD (mg/dL)	0.86 ± 0.15	0.91 ± 0.15	0.079
Preoperative GFR, mean ± SD (ml/dk)	94.17 ± 19.96	84.99 ± 14.99	**0.005**

BC=blood cardioplegia; DM=diabetes mellitus; DNC=Del Nido cardioplegia; GFR=glomerular filtration rate; HT=hypertension; SD=standard deviation

For patients given DNC, mean ACC duration was 56.17 ± 12.03 minutes, mean total pump duration was 93.58 ± 16.54 minutes, mean ICU stay was 50.07 ± 22.54 hours, and mean total hospitalization was 9.18 ± 2.58 days. Mean urea was32.84 ± 8.57 on postoperative first day and 42.24 ± 13.65 on postoperative second day, mean creatinine was 0.87 ± 0.20 on postoperative first day and 0.89 ± 0.35 on postoperative second day, and mean GFR was 94.96 ± 26.59 on postoperative first day and 98.05 ± 34.27 on postoperative second day. Postoperative drainage was 545.83 ± 227.18 ml (first 24 hours) and 268.00 ± 147.46 ml (second 24 hours), and postoperative urine was 2747.50 ± 627.33 ml (first 24 hours) and 2519.67 ± 478.98 ml (second 24 hours). Mean noradrenaline (NA) dose was 0.10 ± 0.08 mcg/kg, and postoperative acute renal injury (ARI) developed in nine cases (15.0%).

In patients with BC, mean ACC duration was 50.77 ± 11.70 minutes, mean total pump duration was 92.13 ± 15.85 minutes, mean ICU stay was 45.43 ± 20.20 hours, and mean total hospitalization was 8.42 ± 1.94 days. Mean urea values were 34.50 ± 9.06 on the postoperative first day and 38.06 ± 12.93 on the postoperative second day, mean creatinine was 0.89 ± 0.14 on the postoperative first day and 0.85 ± 0.17 on the postoperative second day, and mean GFR was 88.00 ± 16.39 on the postoperative first day and 94.57 ± 20.12 on the postoperative second day. For postoperative drainage, values were 552.50 ± 242.15 ml in the first 24 hours and 258.42 ± 121.36 ml in the second 24 hours, with postoperative urine of 3007.03 ± 821.38 ml in the first 24 hours and 2658.50 ± 757.09 ml in the second 24 hours. Mean NA dose was 0.06 ± 0.05mcg/kg with postoperative ARI development in two cases (3.30%).

The ACC duration, ARI development, and amounts of NA were statistically significantly higher in patients with DNC (*P* < 0.05) (Table 2).

**Table 2:** Comparison of some postoperative values of patients according to cardioplegia method.

Features	DNC (n = 60) Mean ± SD	BC (n = 60) Mean ± SD	*P*-value
ACC duration (min)	56.17 ± 12.03	50.77 ± 11.70	**0.014**
Total pump time (min)	93.58 ± 16.54	92.13 ± 15.85	0.625
Length of stay in ICU (hours)	50.07 ± 22.54	45.43 ± 20.20	0.238
Total hospital stay (days)	9.18 ± 2.58	8.42 ± 1.94	0.068
Postoperative first day urea (mg/dL)	32.84 ± 8.57	34.50 ± 9.06	0.454
Postoperative second day urea (mg/dL)	42.24 ± 13.65	38.06 ± 12.93	0.087
Postoperative first day creatinine (mg/dL)	0.87 ± 0.20	0.89 ± 0.14	0.474
Postoperative second day creatinine (mg/dL)	0.89 ± 0.35	0.85 ± 0.17	0.436
Postoperative first day GFR (ml/dk)	94.96 ± 26.59	88.00 ± 16.39	0.087
Postoperative second day GFR (ml/dk)	98.05 ± 34.27	94.57 ± 20.12	0.499
Postoperative drainage (first 24 hours) (ml)	545.83 ± 227.18	552.50 ± 242.15	0.877
Postoperative drainage (second 24 hours) (ml)	268.00 ± 147.46	258.42 ± 121.36	0.698
Postoperative urine (first 24 hours) (ml)	2747.50 ± 627.33	3007.03 ± 821.38	0.054
Postoperative urine (second 24 hours) (ml)	2519.67 ± 478.98	2685.50 ± 757.09	0.154
Postoperative urine by body surface area (first 24 hours)	1496.65 ± 410.90	1592.05 ± 464.87	0.236
Postoperative urine by body surface area (second 24 hours)	1381.19 ± 364.27	1417.74 ± 414.83	0.609
Noradrenaline (mcg/kg) (n = 102)	0.10 ± 0.08 (n = 50)	0.06 ± 0.05 (n = 52)	**0.004**
Postoperative erythrocyte replacement (n = 101)	2.00 (2.00-3.00)	2.00 (1.00-3.00)	0.158
Postoperative AKI development, n (%)	9 (15.00%)	2 (3.30%)	**0.027**

ACC=aortic cross-clamping; AKI=acute kidney injury; BC=blood cardioplegia; DNC=Del Nido cardioplegia; GFR=glomerular filtration rate; ICU=intensive care unit; SD=standard deviation

In patients without DM and with DNC, mean ACC duration was 56.43 ± 13.16 minutes, mean total pump duration was 91.43 ± 17.73 minutes, and mean ICU stay was 47.13 ± 17.93 hours. For postoperative drainage, there were 576.67 ± 238.70 ml in the first 24 hours and 277.67 ± 152.78 ml in the second 24 hours, with postoperative urine amounts of 2701.17 ± 735.29 ml in the first 24 hours and 2464.67 ± 496.69 ml in the second 24 hours. According to body surface area, mean postoperative urine was calculated as 1490.18 ± 462.05 ml in the first 24 hours and 1365.67 ± 358.22 ml in the second 24 hours.

For patients without DM and with BC, the mean ACC duration was 49.78 ± 10.13 minutes with mean total pump duration of 95.03 ± 15.60 minutes and mean ICU stay of 46.13 ± 18.80 hours. Postoperative drainage was 603.91 ± 260.66 ml in the first 24 hours and 251.72 ± 125.81 ml in the second 24 hours. Postoperative urine was 2960.84 ± 708.70 ml in the first 24 hours and 2646.56 ± 838.74 ml in the second 24 hours. When calculated according to body surface area, postoperative urine amounts were 1547.48 ± 400.22 ml in the first 24 hours and 1382.91 ± 459.88 ml in the second 24 hours.

Among patients without DM and with DNC, the ACC duration was statistically significantly higher compared to the patient group with BC (*P* = 0.029) (Table 3).

**Table: 3:** Comparison of some values according to cardioplegia method in patients with and without DM.

	Comparison of some values according to the cardioplegia method in patients without DM			Comparison of some values according to cardioplegia method in patients with DM		
Features	DNC (n = 30)	BC (n = 32)	*P*-value	DNC (n = 30)	BC (n = 28)	*P*-value
	Mean ± SD	Mean ± SD		Mean ± SD	Mean ± SD	
ACC duration (min)	56.43 ± 13.16	49.78 ± 10.13	**0.029**	55.90 ± 11.00	51.89 ± 13.37	0.217
Total pump time (min)	91.43 ± 17.73	95.03 ± 15.60	0.399	95.73 ± 15.26	88.82 ± 15.76	0.095
Length of stay in ICU (hours)	47.13 ± 17.93	46.13 ± 18.80	0.830	53.00 ± 26.35	44.64 ± 22.01	0.197
Total hospital stay (days)	8.97 ± 2.28	8.13 ± 1.94	0.123	9.40 ± 2.87	8.75 ± 1.91	0.313
Postoperative drainage (first 24 hours) (ml)	576.67 ± 238.70	603.91 ± 260.66	0.670	515.00 ± 214.61	493.75 ± 208.34	0.704
Postoperative drainage (second 24 hours) (ml)	277.67 ± 152.78	251.72 ± 125.81	0.467	258.33 ± 143.88	266.07 ± 117.89	0.824
Postoperative urine (first 24 hours) (ml)	2701.17 ± 735.29	2960.84 ± 708.70	0.162	2793.83 ± 505.66	3059.82 ± 944.56	0.193
Postoperative urine (second 24 hours) (ml)	2464.67 ± 496.69	2646.56 ± 838.74	0.307	2574.67 ± 462.38	2730.00 ± 664.05	0.303
Postoperative urine by body surface area (first 24 hours)	1490.18 ± 462.05	1547.48 ± 400.22	0.603	1503.12 ± 360.45	1642.99 ± 532.23	0.250
Postoperative urine by body surface area (second 24 hours)	1365.67 ± 358.22	1382.91 ± 459.88	0.870	1396.71 ± 375.69	1457.54 ± 360.74	0.532

ACC=aortic cross-clamping; BC=blood cardioplegia; DM=diabetes mellitus; DNC=Del Nido cardioplegia; ICU=intensive care unit; SD=standard deviation

In patients receiving and not receiving preoperative autologous blood, there were no statistically significant differences between urea, creatinine, and GFR on the postoperative first and second days, or with ICU duration, total hospitalization, and postoperative second 24-hour drainage amounts (*P* > 0.05). However, in terms of postoperative first 24-hour drainage amounts, patients administered preoperative autologous blood had mean drainage amounts that were statistically significant compared to the mean drainage amounts of patients not administered preoperative autologous blood (*P* < 0.05) (Table 4).

**Table 4:** Comparison of some values of the patients according to autologous blood collection status.

Features	Not Autologous (n = 37)	Autologous Received (n = 83)	*P*-value
	Mean ± SD	Mean ± SD	
Postoperative first day urea (mg/dL)	35.23 ± 9.21	32.64 ± 8.55	0.139
Postoperative second day urea (mg/dL)	40.43 ± 11.92	40.03 ± 14.08	0.879
Postoperative first day creatinine (mg/dL)	0.86 ± 0.17	0.89 ± 0.18	0.390
Postoperative second day creatinine (mg/dL)	0.88 ± 0.34	0.86 ± 0.24	0.750
Postoperative first day GFR (ml/dk)	88.54 ± 21.48	92.79 ± 22.62	0.337
Postoperative second day GFR (ml/dk)	89.25 ± 24.38	99.46 ± 29.10	0.065
Length of stay in ICU (hours)	51.14 ± 29.27	46.24 ± 16.83	0.347
Total hospital stay (days)	9.27 ± 2.29	8.59 ± 2.29	0.137
Postoperative drainage (first 24 hours) (ml)	475.68 ± 210.94	581.93 ± 237.24	**0.021**
Postoperative drainage (second 24 hours) (ml)	250.68 ± 124.23	268.80 ± 139.28	0.498

GFR=glomerular filtration rate; ICU=intensive care unit; SD=standard deviation

Patients not administered autologous blood did not have any statistically significant differences for postoperative first and second day urea, creatinine, GFR, total hospitalization, and postoperative first 24 hours and second 24 hours drainage when DNC and BC groups were compared (*P* > 0.05). Between both cardioplegia methods, patients in the DNC group had statistically significantly longer ICU duration (*P* = 0.006) (Table 5). Assessment according to DM patients found no statistically significant difference for ARI development and ARI stages in terms of patient numbers between patients with DM (n = 58) and patients without DM (n = 62) (*P* > 0.05) (Table 6).

**Table 5:** Some values of patients in whom autologous blood was not taken according to the cardioplegia method comparison.

Features	DNC (n = 23)	BC (n = 14)	*P*-value
	Mean ± SD	Mean ± SD	
Postoperative first day urea (mg/dL)	35.07 ± 10.35	35.47 ± 7.31	0.900
Postoperative second day urea (mg/dL)	41.92 ± 12.04	37.98 ± 11.73	0.337
Postoperative first day creatinine (mg/dL)	0.85 ± 0.17	0.87 ± 0.19	0.817
Postoperative second day creatinine (mg/dL)	0.91 ± 0.40	0.84 ± 0.21	0.551
Postoperative first day GFR (ml/dk)	89.78 ± 24.27	86.51 ± 16.56	0.660
Postoperative second day GFR (ml/dk)	87.61 ± 25.53	91.93 ± 23.03	0.608
Length of stay in ICU (hours)	61.04 ± 28.94	34.86 ± 22.28	**0.006**
Total hospital stay (days)	9.78 ± 2.27	8.43 ± 2.13	0.081
Postoperative drainage (first 24 hours) (ml)	502.17 ± 230.35	432.14 ± 173.60	0.334
Postoperative drainage (second 24 hours) (ml)	257.61 ± 143.30	239.29 ± 88.09	0.633

BC=blood cardioplegia; DNC=Del Nido cardioplegia; GFR=glomerular filtration rate; ICU=intensive care unit; SD=standard deviation

**Table 6:** Comparison of AKIN development status in patients according to diabetes mellitus status.

Acute kidney failure (AKIN)	Yes	5 (8.60%)	6 (9.70%)	0.841
	No	53 (91.40%)	56 (90.30%)	
	Total	58 (100%)	62 (100%)	
Acute kidney failure stage (AKIN)	Stage1	4 (80.0%)	4 (66.70%)	1.000*
	Stage2	1 (20.0%)	2 (33.30%)	
	Total	5 (100%)	6 (100%)	

*Fisher’s exact chi-square test was used AKIN=Acute Kidney Injury Network

## DISCUSSION

BC solution was defined by Buckberd in 1979 and led the way to significant developments in myocardial protection^[8]^ Due to BC solution being administered multiple times, relatively difficult preparation, and cost effectiveness reasons, the search for other cardioplegia solutions began. DNC was reported to provide good and safe myocardial protection and has less cost compared to BC^[9^, ^10]^. Lengthened ACC duration is associated with mortality and cardiac and renal function disorders in the postoperative period. For this reason, implementing precautions about reducing the ACC duration may be helpful to improve outcomes^[11^, ^12]^. While there may be differences in cardioplegia and practices between clinics even though surgical procedures are the same, there are studies showing that single-dose cardioplegia has better reliability compared to multiple dose administration and that cardiopulmonary bypass (CPB) and ACC durations are reduced more^[13]^. A study by Akar in 2022 reported that there was no statistically significant difference identified in a comparison of ACC and CPB durations between patients with DNC and BC used^[14]^.

In our study comparing DNC and BC, patients with DNC had significantly higher ACC duration compared to patients with BC (*P* = 0.014) (Table 2). It is thought this may be linked to limiting external factors (different team, different surgeon, different operating rooms, and limited number of patients). However, there was no statistically significant difference identified in terms of total pump duration between the two cardioplegia methods (*P* > 0.05). When DNC and BC administration was compared in patients receiving autologous blood, no significant difference was found (*P* > 0.05). No significant differences were identified between DNC and BC among patients with DM (*P* > 0.05). The ACC duration in patients with DNC, among patients without DM, was significantly longer than the mean ACC duration for patients with BC (*P* = 0.029) (Table 3). A study by Kuserli et al.^[15]^ related to aortic root surgery reported no difference in terms of inotrope doses administered to the two groups. However, in our study, the mean NA dose administered to patients with DNC was significantly higher than the mean NA dose administered to patients with BC (*P* = 0.004) (Table 2). Pourmoghadam KK et al.^[16]^, in the comparison of DNC and BC, reported that there was no significant difference considering the analysis of repeated measurements for the average measured quantities and measurement values. Similarly, in our study, no statistically significant difference was identified between postoperative urine amounts and postoperative urine amounts according to body surface area (*P* > 0.05). The comparison of patients who did not receive autologous blood according to cardioplegia method found that ICU durations were statistically significantly longer in patients administered DNC (*P* = 0.006) (Table 5). Independently of cardioplegia method, mean cumulative assessment of postoperative first 24-hour drainage of patients receiving autologous blood found significantly higher values compared to mean drainage in patients not receiving autologous blood (*P* = 0.012) (Table 4). ARI and CRF developing after lengthened ACC and CPB durations in open heart surgery are associated with increasing morbidity and both short- and long-term mortality rates^[6^, ^7]^. For assessment of ARI and CRF, urea and creatinine levels are important parameters indicating renal functions^[17^, ^18]^. When urea and creatinine levels in blood are evaluated, no significant difference was detected between patients administered DNC and BC solutions^[19]^. In our study when urea and creatinine levels are compared according to cardioplegia method, no statistically significant difference was found, similar to previous studies (*P* > 0.05) (Table 2). A study by Tan et al.^[20]^ identified no difference in terms of renal failure, duration in the ICU, and total hospitalization when two different cardioplegia solutions were used^[21]^. In our study, there was no significant difference in GFR levels between the two cardioplegia solutions (*P* = 0.499). However, when ARI status is compared according to the cardioplegia method used, patients with BC had less ARI development, and this was statistically significant (*P* = 0.027) (Table 2). This situation is thought to be due to the longer ACC duration in patients with DNC. When postoperative erythrocyte replacement counts are compared, no statistically significant difference was identified between the two groups (*P* > 0.05). Bişar et al.^[22]^ stated there were no statistically significant differences for duration in intensive care and total hospitalization between DNC and BC groups. Similarly, in our study, there were no statistically significant differences identified between the two groups in terms of intensive care duration and total hospitalization (*P* > 0.05).

In the cases included in our study, no cerebrovascular event was experienced during the in-hospital stay and there was no mortality.

### Limitations

Limitations of our study are the different surgical teams, different surgeons, single-center setting, being retrospective, and including low numbers of patients. The inotropes, diuretics, antibiotics, and antihypertensive drugs administered to patients in the postoperative period may have statistically impacted the urea, creatinine, and urine amounts of patients. Additionally, assessment of cystatin C, a more sensitive and important kidney function marker, and similar parameters was not performed in our study.

## CONCLUSION

In our study, DNC use lengthened the ACC duration, increased NA needs, and more ARF occurred compared to BC. There is a need for broader meta-analysis with minimized limitations and studies researching more sensitive renal function measurement parameters in relation to the DNC solution administered during adult heart surgery in this study.

## Abbreviations, Acronyms & Symbols

ACC: Aortic cross-clamping
AKI: Acute kidney injury
AKIN: Acute Kidney Injury Network
ARF: Acute renal failure
ARI: Acute renal injury
BC: Blood cardioplegia
BUN: Blood urine nitrate
CPB: Cardiopulmonary bypass
DM: Diabetes mellitus
DNC: Del Nido cardioplegia
GFR: Glomerular filtration rate
HT: Hypertension
ICU: Intensive care unit
NA: Noradrenaline
SD: Standard deviation
